# Effect of Omega-3 Fatty Acid Supplementation on Plasma Fibroblast Growth Factor 23 Levels in Post-Myocardial Infarction Patients with Chronic Kidney Disease: The Alpha Omega Trial

**DOI:** 10.3390/nu9111233

**Published:** 2017-11-11

**Authors:** Martin H. de Borst, Leandro C. Baia, Ellen K. Hoogeveen, Erik J. Giltay, Gerjan Navis, Stephan J. L. Bakker, Johanna M. Geleijnse, Daan Kromhout, Sabita S. Soedamah-Muthu

**Affiliations:** 1 Department of Nephrology, University of Groningen, University Medical Center Groningen, Hanzeplein 1, 9713 GZ Groningen, The Netherlands; leandronut@yahoo.com.br (L.C.B.); g.j.navis@umcg.nl (G.N.); s.j.l.bakker@umcg.nl (S.J.L.B.); 2 Department of Nephrology, UNIFESP, Rua Botucatu, 740, Vila Clementino, São Paulo 04023900, SP, Brazil; 3 Department of Internal Medicine and Nephrology, Jeroen Bosch Hospital, Henri Dunantstraat 1, 5223 GZ Den Bosch, The Netherlands; e.k.hoogeveen@lumc.nl; 4 Department of Clinical Epidemiology, Leiden University Medical Center, Albinusdreef 2, 2333 ZA Leiden, The Netherlands; 5 Department of Psychiatry, Leiden University Medical Center, Albinusdreef 2, 2333 ZA Leiden, The Netherlands; e.j.giltay@lumc.nl; 6 Division of Human Nutrition, Wageningen University & Research, Stippeneng 4, 6708 WE Wageningen, The Netherlands; marianne.geleijnse@wur.nl (J.M.G.); daan.kromhout@wur.nl (D.K.); sabita.soedamah-muthu@wur.nl (S.S.S.-M.)

**Keywords:** n-3 polyunsaturated fatty acids, fibroblast growth factor 23, myocardial infarction, chronic kidney disease, cardiovascular

## Abstract

Fibroblast growth factor 23 (FGF23) is an independent risk factor for cardiovascular mortality in chronic kidney disease. Omega-3 (n-3) fatty acid consumption has been inversely associated with FGF23 levels and with cardiovascular risk. We examined the effect of marine n-3 fatty acids eicosapentaenoic acid (EPA) and docosahexaenoic acid (DHA) and plant-derived alpha-linolenic acid (ALA) on plasma FGF23 levels in post-myocardial infarction patients with chronic kidney disease. In the randomized double-blind Alpha Omega Trial, 4837 patients with a history of myocardial infarction aged 60–80 years (81% men) were randomized to one of four trial margarines supplemented with a targeted additional intake of 400 mg/day EPA and DHA, 2 g/day ALA, EPA-DHA plus ALA, or placebo for 41 months. In a subcohort of 336 patients with an eGFR < 60 mL/min/1.73 m^2^ (creatinine-cystatin C-based CKD-EPI formula), plasma C-terminal FGF23 was measured by ELISA at baseline and end of follow-up. We used analysis of covariance to examine treatment effects on FGF23 levels adjusted for baseline FGF23. Patients consumed 19.8 g margarine/day on average, providing an additional amount of 236 mg/day EPA with 158 mg/day DHA, 1.99 g/day ALA or both, in the active intervention groups. Over 79% of patients were treated with antihypertensive and antithrombotic medication and statins. At baseline, plasma FGF23 was 150 (128 to 172) RU/mL (mean (95% CI)). After 41 months, overall FGF23 levels had increased significantly (*p* < 0.0001) to 212 (183 to 241) RU/mL. Relative to the placebo, the treatment effect of EPA-DHA was indifferent, with a mean change in FGF23 (95% CI) of −17 (−97, 62) RU/mL (*p* = 0.7). Results were similar for ALA (36 (−42, 115) RU/mL) and combined EPA-DHA and ALA (34 (−44, 113) RU/mL). Multivariable adjustment, pooled analyses, and subgroup analyses yielded similar non-significant results. Long-term supplementation with modest quantities of EPA-DHA or ALA does not reduce plasma FGF23 levels when added to cardiovascular medication in post-myocardial patients with chronic kidney disease.

## 1. Introduction

Chronic kidney disease (CKD) affects 8–16% of the global population and approximately 30% of patients requiring percutaneous coronary intervention for acute coronary syndrome or stable angina [1,2]. Cardiovascular disease is by far the leading cause of death in CKD patients [3]. The mechanisms underlying the high cardiovascular risk in CKD are incompletely understood. Although classical cardiovascular risk factors including diabetes and hypertension are more prevalent in CKD than in the general population, these factors do not explain all of the excess cardiovascular risk in these patients [4,5]. Deregulated calcium/phosphate homeostasis promotes vascular calcification, which in turn is considered to substantially contribute to the premature development and rapid progression of cardiovascular disease in individuals with and without CKD [6,7]. Specifically, recent epidemiological studies have linked a high plasma level of the phosphaturic hormone fibroblast growth factor 23 (FGF23) with an increased risk of cardiovascular disease in CKD patients [8,9,10,11]. A higher FGF23 level has also been associated with an impaired response to intensified renin–angiotensin–aldosterone system (RAAS)-blockade-based therapy, the key therapy in patients with both kidney and cardiovascular disease [12].

In order to explore whether FGF23 reduction could provide cardiovascular protection in CKD patients, novel strategies to lower plasma FGF23 levels are needed. We recently found that in kidney transplant recipients, n-3 fatty acid intake was inversely associated with FGF23 levels, independent of known determinants of FGF23 levels [13]. Furthermore, supplementation with omega-3 (n-3) fatty acids provided a beneficial effect on kidney function in patients with a history of myocardial infarction [14], which may in turn reduce plasma FGF23 level. These observations may be reconciled with a randomized trial showing cardiovascular protective effects of n-3 fatty acids in the CKD population [15]. On the other hand, an open-label controlled trial in 47 hemodialysis patients with relatively short follow-up (six months) was unable to demonstrate an effect of n-3 supplementation on plasma FGF23 levels [16]. 

We addressed whether intervention with dietary n-3 fatty acid supplementation for 41 months may reduce plasma FGF23 levels in post-myocardial infarction patients participating in the Alpha Omega Trial, a large randomized controlled trial that compared treatment with low-dose eicosapentaenoic acid-docosahexaenoic acid (EPA-DHA) or alpha-linoleic acid (ALA) supplementation with placebo [17]. The current study was performed in a subgroup of patients with chronic kidney disease stage 3 (estimated glomerular filtration rate (eGFR) < 60 mL/min/1.73 m^2^).

## 2. Materials and Methods

### 2.1. Design of the Alpha Omega Trial

**Figure 1:**
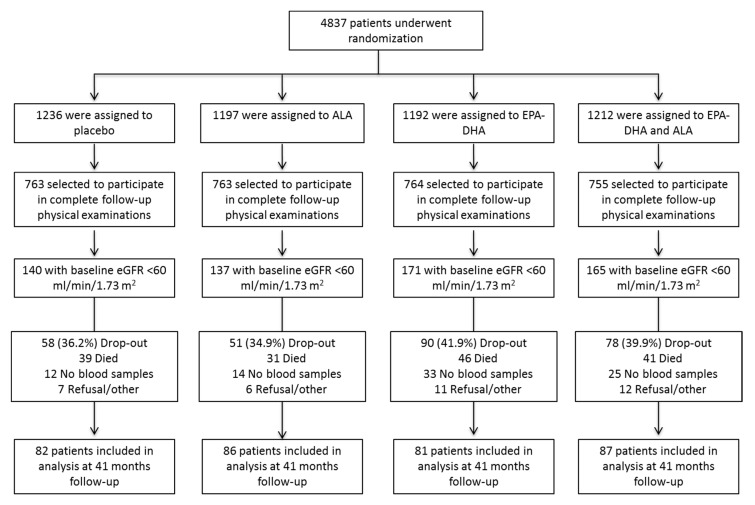
Flowchart of the FGF23 biomarker study within the Alpha Omega Trial. Participant flow for the study on the effect of n-3 fatty acid supplementation on plasma C-terminal FGF23 levels in post myocardial infarction patients in the Alpha Omega Trial with chronic kidney disease stage 3(eGFR < 60 mL/min/1.73 m^2^).

The present study used data from the Alpha Omega Trial, a multicenter, randomized, double-blind, placebo-controlled trial conducted between 2002 and 2009 (ClinicalTrials.gov no. NCT00127452). Details of the trial have been reported previously [17,18]. In brief, the study population consisted of 4837 patients with a verified history of myocardial infarction living in The Netherlands, who were 60–80 years old upon inclusion, and received state-of-the-art antihypertensive medication, antithrombotic medication, and statins. Patients were recruited sequentially and randomly assigned to receive one of four trial margarines (20 g daily) for 41 months. Actual treatment was for logistic reasons preceded by 4–6 weeks on placebo margarine. The trial margarines were identical except for their n-3 fatty acid content, and provided a targeted additional daily intake of 400 mg EPA-DHA (ratio of 3:2), a targeted additional daily intake of 2 g of ALA, a combination of EPA-DHA and ALA, or placebo. Dosages were comparable with the Recommended Dietary Allowances (RDA) for EPA+DHA (400 mg/day) [19] and ALA (±2 g/day) [20,21]. Patients were asked to refrain from n-3 fatty acid supplements during participation in the trial. Compliance to the trial margarines was monitored by counting of margarine tubs, telephone interviews, and patient diaries. Trained research staff performed structured telephone interviews at 12 and 24 months after the start of the intervention, to collect data on adherence, cardiovascular events, adverse events, changes in medication, intake of fish, and use of n-3 fatty acid supplements. The present study was limited to patients randomized before August 2005 (Figure 1).

For the present study, patients with chronic kidney disease stage 3, defined as an estimated GFR (eGFR) <60 mL/min/1.73 m^2^ were selected. The eGFR was calculated using the combined creatinine-cystatin C-based CKD-EPI formula from 2012 [22]. Blood samples were drawn at baseline and after 41 months of follow-up. The Alpha Omega Trial was conducted in accordance with the Declaration of Helsinki and approved by a central medical ethics committee in the Netherlands (Office for Human Research Protections #IORG000404). Written informed consent was obtained from all patients.

### 2.2. Laboratory Measurements

Standardized blood handling procedures were previously described in detail [23]. Briefly, blood samples were obtained in the morning at the participant’s home or hospital. Subsequently, tubes were packaged in sealed envelopes and sent by standard postal service to a central laboratory. We asked subjects to remain fasted if physical examination was scheduled before 10:30 a.m. or to consume only a light meal when the examination was scheduled after 10:30 a.m. and to refrain from smoking or drinking coffee 1 h before blood sampling.

At baseline and at 41 months of follow-up, creatinine and cystatin C were measured from stored serum samples in a central laboratory. Serum creatinine was measured by the modified kinetic Jaffé method (Dimension Vista 1500 Analyzer; Siemens, Munich, Germany). We calibrated directly to the standard supplied by the manufacturer from the National Institute of Standards and Technology Standard Reference Material, and postcalibration correction was applied [24]. Intra- and interassay variations for low creatinine (mean = 71 mmol/L) were 1.8% and 2.9%, respectively, and for high creatinine (mean = 345 mmol/L), 0.8% and 2.2%, respectively. Serum creatinine values < 53 mmol/L were unreliable (owing to technical failure or analytical disturbance; *n* = 82) and therefore not reported in accordance with the Standard Operating Procedure of the central laboratory. Serum cystatin C was measured using a particle-enhanced immunonephelometric assay (N Latex Cystatin C, Dimension Vista 1500 Analyzer; Siemens), as described previously [14]. Calibrators and assays of the same lot code were used, which was stable. Cystatin C was calibrated directly using the standard supplied by the manufacturer [25]. The analytical measurement range of cystatin C was 0.23–8.00 mg/L. Intra- and interassay variation coefficients for low cystatin C (1.00 mg/L) were 1.3% and 4.2%, respectively. These variation coefficients for high cystatin C (1.75 mg/L) were 2.9% and 2.8%, respectively. Intra- and interassay variations were 0.8% and 2.2%, respectively. Plasma C-terminal FGF23 levels were determined at baseline and at 41 months of follow-up by sandwich ELISA (Immutopics, San Clemente, CA, USA), with intra-assay and interassay coefficients of variation of <5% and <16%, respectively [26]. High-sensitivity C-reactive protein (hsCRP) levels were measured in stored serum samples (Nephelometric, Dimension Vista 1500 analyzer, Siemens). Intra- and inter-assay variation for low hsCRP (mean 1.0 mg/L) was 2.1% and 1.6%, and for high hsCRP (mean 2.8 mg/L) 2.0% and 2.4%, respectively [27]. To quantify compliance with the study margarines, fatty acids (percent weight) were measured in plasma cholesteryl esters using the method described by Glatz et al. [28].

### 2.3. Demographic and Clinical Data Collection

Patients were interviewed and physically examined by trained research nurses at home or in the hospital. Information on demographic variables, lifestyle habits, current health status, and medical history was collected by self-administered questionnaires as previously described in detail [18]. Ethnicity was categorized as white, black, or other. Medication was coded according to the Anatomical Therapeutic Chemical Classification System. Diabetes was defined as self-reported physician diagnosis, antidiabetic medication (including insulin) or by plasma glucose concentrations (≥7 mmol/L (126 mg/dL) for those fasting and ≥11.1 mmol/L (200 mg/dL) for non-fasting patients) [29].

## 3. Statistical Analyses

A pre-specified statistical analysis plan was used for these analyses. Based on data from a previous study [13,30], we calculated a required sample size of 59 per group to detect a 30% reduction in log-transformed FGF23 with 80% power and an alpha of 0.05. Baseline descriptors of the four treatment groups were presented as mean ± SD, median with interquartile range (IQR) or percentage. Intention-to-treat analyses were performed. The plasma FGF23 concentrations at 41 months of treatment, adjusted for the baseline FGF23 levels, were analyzed using analysis of covariance (ANCOVA) models with treatment as independent variable based on a previous publication [31]. Log-transformations were used if absence of normality. The *p*-value for equal error variances (Levene’s test if >0.05) indicates whether the error variance is equal across the treatment group, i.e., whether the assumption of equal error variances is met and ANCOVA is the correct method for comparing the groups. The primary objective was to compare FGF23 concentrations at the end of follow-up between the EPA-DHA group vs. the placebo group. As secondary analyses, we studied the effects of ALA vs. placebo and the combination of EPA-DHA and ALA vs. placebo on plasma concentrations of FGF23. Further adjustments for several covariates (age, sex, cigarette smoking, alcohol intake, education level, prevalent diabetes, physical activity status and high-sensitivity C-reactive protein (hsCRP) levels) were performed. Spearman correlation coefficients were calculated for the relationship between baseline and follow-up FGF23, and for the relationship between FGF23 and eGFR and hsCRP, respectively. Paired *t*-tests were performed to check whether differences between baseline post-treatment were statistically significant. Subgroup analyses were performed according to the presence of diabetes mellitus, obesity, or both at baseline. Analyses were repeated after exclusion of subjects with extreme FGF23 values at baseline. As a secondary analysis we analyzed the intervention groups according to the 2 × 2 factorial design as in the original paper [17], retaining statistical power by keeping all treatments groups in the analyses and less between-treatment comparisons then the initially used method. We also performed sensitivity analyses stratified for protein intake (above vs. below the median), since protein intake in itself may influence FGF23 [32], and for renin-angiotensin-aldosterone system (RAAS) blocker use, given potential cross-talk between FGF23 and the RAAS [33]. Fish oil supplements were used by a too small number of patients (*N* = 16) to perform stratified analyses. All analyses were performed blind to the intervention code. Statistical analysis was carried out using SPSS version 20.0 (SPSS, Inc., Chicago, IL, USA). Two-sided *p* values < 0.05 were regarded as statistically significant.

## 4. Results

### 4.1. Characteristics of the Study Population

Our study population consisted of 336 patients with a mean age of 73.0 years (SD 4.9), 68.8% were males, 30.1% had obesity, 27.7% had type 2 diabetes and 13.7% were current cigarette smokers. The median period between the index myocardial infarction and entry into the study was 4.0 years (IQR, 2.0 to 6.3). Antihypertensive and antithrombotic medication was used by 95.2% and 95.8% of all patients, and statins were used by 79.5%. Median intake of EPA-DHA was 93.0 (IQR: 37.9–166.3) mg/day. The median daily fish intake was 10.8 (IQR: 4.1, 18.3) g and 28% of all patients had a fish intake below 5 g/day. Baseline characteristics of the study population by n-3 treatment groups are shown in Table 1. Baseline characteristics, including FGF23, were well balanced over the four study groups.

### 4.2. Intake of n-3 Fatty Acids

Ninety-six percent of all patients used margarines >80% of the time and the mean intake of margarines during the trial was 19.7 (SD 4.0) g/day proving an additional amount of 236 mg/day EPA with 158 mg/day DHA, 1.99 g/day ALA or both, in the active treatment groups. Fish oil capsules or supplementation was used by 16 (5.0%) patients, with a similar distribution over all treatment groups.

### 4.3. Effect of n-3 Fatty Acids on Plasma FGF23 Levels

**Figure 2:**
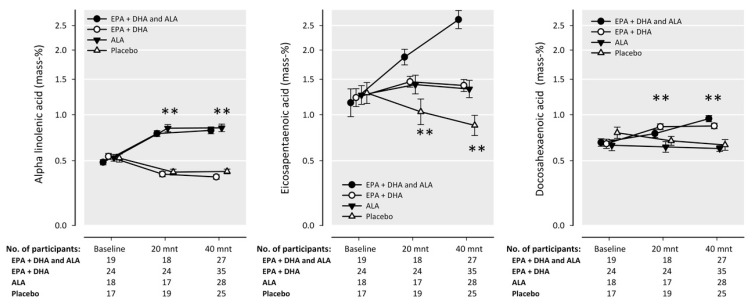
The effect of supplementation of n-3 fatty acids in margarines on serum cholesteryl ester biomarkers within Alpha Omega Trial participants included in the current analysis. Geometric mean values (expressed as mass percentage) with error bars indicating 95% confidence intervals, on a logarithmic scale. ** *p* < 0.01 between groups.

Patients participated in the trial for a median of 41.4 (IQR: 40.8, 42.0) months. At baseline, plasma FGF23 was 150 (128 to 172) RU/mL (mean (95% CI)). After 41 months, overall FGF23 levels had increased significantly (*p* < 0.0001) to 212 (183 to 241) RU/mL. The Spearman correlation between baseline and follow-up FGF23 concentrations was 0.71, *p* < 0.0001. The effects of different n-3 fatty acids intervention groups on FGF23 levels are presented in Table 2, all relative to placebo. Achieved FGF23 levels, adjusted for baseline FGF23 levels, were similar across all intervention groups. Relative changes in FGF23 were minor and not statistically significant (all *p* > 0.3) in the comparisons for EPA-DHA, ALA, EPA-DHA + ALA vs. placebo. The change in plasma FGF23 levels over time was inversely correlated with the change in eGFR over 41 months (spearman correlation coefficient = −0.42, *p* < 0.0001). Measurements of n-3 fatty acids in plasma cholesteryl esters (Figure 2) indicated sufficient n-3 intake throughout the study. During follow-up, eGFR decreased from 47.4 (SD 9.9) to 44.6 (14.0) mL/min/1.73 m^2^ (*p* < 0.0001). The change in plasma FGF23 levels was positively correlated with the change in hsCRP over 41 months (spearman correlation coefficient = 0.16, *p* = 0.003). Median hsCRP was 2.9 (IQR: 1.4, 5.5) at baseline and 3.4 (1.4, 7.3) at the end of follow-up (*p* = 0.07).

Secondary analyses using the 2 × 2 factorial design approach (two-way ANOVA) comparing EPA-DHA vs. placebo/ALA and ALA vs. EPA-DHA/placebo showed similar null effects (Table 3). Analyses by subgroups (prevalent diabetes, obesity or a combination of the two conditions) also demonstrated no effects of any of the types of n-3 fatty acid supplementation on plasma FGF23 levels over a 40-month period (data not shown). The results were similar when patients with extreme FGF23 values (>600 RU/mL) were excluded (*n* = 22). 

Sensitivity analyses stratified for protein intake (above vs. below the mean protein intake of 0.8 g/kg body weight) or renin–angiotensin–aldosterone system blocker use yielded results similar to the primary analysis.

## 5. Discussion

The main finding of this study is that in post-myocardial infarction patients with chronic kidney disease (eGFR < 60 mL/min/1.73 m^2^), low dose n-3 fatty acid supplementation did not reduce plasma C-terminal FGF23 levels. To our knowledge, this is the first study with long-term follow-up to prospectively assess the effect of n-3 fatty acids on circulating FGF23 levels. Cardiovascular disease is the leading cause of death in patients with chronic kidney disease. The rationale for the current study was that on the one hand FGF23 has been robustly associated with an increased cardiovascular risk in CKD patients, providing a rationale for prospective intervention studies targeting plasma FGF23, and on the other hand our recent finding that higher n-3 fatty acid intake is independently associated with lower plasma C-terminal FGF23 levels, albeit in a different patient population (renal transplant recipients) [13].

When we identified n-3 fatty acid intake as an independent determinant of FGF23 levels in our previous study, we hypothesized that the anti-inflammatory effects of n-3 fatty acids would influence renal expression of klotho, a major cofactor for the FGF23 receptor [34]. Accordingly, several publications have now linked an inflammatory state with higher levels of FGF23 [35,36]. In vitro, n-3 fatty acids are able to reduce inflammation in tubular epithelial cells [37], and also reduce oxidative stress [38], another factor regulating renal klotho expression in vitro and in vivo [39,40]. Whether low dose n-3 supplementation (comparable with the RDA) used in the Alpha Omega Trial was capable of reducing renal inflammation or oxidative stress in this subcohort with CKD stage 3 is unclear. A secondary analysis of the entire Alpha Omega Trial population demonstrated no effect of n-3 fatty acid supplementation on circulating hsCRP, reflecting systemic inflammation [27]. Although several other randomized controlled trials used a much higher dose of n-3 fatty acids (1–6 grams/day), these studied also did not show a significant effect on circulating parameters of inflammation [41,42,43], although this does not exclude an effect on intra-renal inflammation, which may have remained undetected by circulating markers. In the current study we found a significant association between FGF23 and hsCRP at the individual patient level, in line with our hypothesis and a previous study [35].

Our findings are in line with a previous open label study in 47 hemodialysis patients treated with n-3 fatty acids or control treatment for six months, in which no effect was observed on plasma FGF23 levels [16]. Likely, the decrease in eGFR over time in our cohort of patients with CKD at baseline was a major driver towards increased FGF23, which may have counteracted a potential FGF-23 lowering effect by n-3 fatty acid supplementation. Possibly a higher dose of n-3 fatty acids might be able to induce a stronger effect on kidney function, plasma FGF23 levels, or both.

Our study has several strengths and limitations. Strengths include the relatively long follow-up (41 months), the centrally analyzed cystatin C and FGF23 measurements, the good compliance to n-3 fatty acid supplementation, the balanced randomization and balanced drop-out rates among the treatment arms, and the n-3 fatty acids doses that are comparable to the recommended daily allowance and thus the real-life situation. The lack of a dose-response design is a limitation of our study. Moreover, we could not measure other parameters of phosphate metabolism including serum phosphate, vitamin D, and parathyroid hormone. Patients were already treated with the state-of-the-art post-myocardial infarction medical therapy including antihypertensive, antithrombotic medication and statins, which may have diluted the treatment effect. The dose of n-3 fatty acid supplementation was relatively low; yet sufficient to change fatty acid composition of cholesteryl esters, which is considered a valid biomarker for intake of n-3 fatty acids in the past weeks [44]. Another limitation is that the population of our current study, elderly subjects with cardiovascular history and CKD, differs in several aspects from the renal transplant population in which we initially identified the association between n-3 fatty acid intake and FGF23 levels [13], among others in terms of cardiovascular history and medication use (particularly immunosuppressive medication), although renal function was comparable among both studies. The relatively high dropout rate may have also influenced our results. Finally, we could not use fasting blood samples in 48% of patients, which could have influenced the results; although differential associations with outcomes have been associated for fasting vs non-fasting serum phosphorus levels, this is unknown for FGF23 levels [45]. Our prior observation urges a confirmatory intervention study to address the effect of n-3 fatty acids in kidney transplant recipients. 

## 6. Conclusions

Our data do not support n-3 fatty acid supplementation to reduce FGF23 levels in post-myocardial infarction patients with CKD. Whether n-3 fatty acids, particularly when given in a higher dose, affect FGF23 levels in other populations remains to be addressed in future prospective studies.

## Figures and Tables

**Table 1:** Baseline characteristics of 336 patients of the Alpha Omega Trial by treatment group with creatinine–cystatin C-based eGFR< 60 mL/min/1.73 m^2^.

	EPA-DHA and ALA (*n* = 87)	EPA-DHA (*n* =8 1)	ALA (*n* = 86)	Placebo (*n* = 82)
Age (y)	73.0 ± 5.0	73.5 ± 4.7	73.1 ± 4.8	72.2 ± 4.8
BMI (kg/m^2^)	28.2 ± 4.6	27.7 ± 4.3	28.7 ± 4.1	28.1 ± 4.1
Time since MI (y)	4.7 ± 3.0	4.4 ± 3.6	4.7 ± 2.8	4.2 ± 3.0
Systolic BP (mmHg)	144.3 ± 25.6	141.5 ± 22.7	145.6 ± 20.7	143.6 ± 24.0
Diastolic BP (mmHg)	79.1 ± 10.8	78.7 ± 11.1	77.5 ± 11.3	78.1 ± 11.3
Glucose (mmol/L)	5.8 ± 1.4	5.9 ± 1.7	6.6 ± 2.6	6.3 ± 2.0
Total serum cholesterol (mmol/L)	4.9 ± 1.1	4.9 ± 1.2	5.0 ± 1.1	5.1 ± 0.9
LDL-cholesterol (mmol/L)	2.7 ± 0.8	2.8 ± 1.0	2.8 ± 1.0	2.9 ± 0.8
HDL-cholesterol (mmol/L)	1.2 ± 0.3	1.3 ± 0.4	1.2 ± 0.3	1.2 ± 0.3
Triglycerides (mmol/L)	1.9 (1.3, 2.5)	1.8 (1.4, 2.2)	2.0 (1.5, 2.6)	1.9 (1.5, 2.6)
Protein intake (g/kg body weight)	0.80 ± 0.23	0.81 ± 0.27	0.79 ± 0.22	0.81 ± 0.24
Fish intake (g/day)	9.9 (1.4, 17.5)	9.7 (4.2, 18.3)	11.0 (4.6, 18.2)	15.0 (5.0, 22.7)
EPA + DHA (mg/day)	76.0 (33.9, 154.1)	69.1 (25.5, 150.8)	98.8 (42.6, 166.6)	114.9 (51.7, 194.2)
Serum cystatin C (mg/L)	1.4 ± 0.3	1.4 ± 0.2	1.4 ± 0.3	1.4 ± 0.4
Serum creatinine (µmol/L)	132.5 ± 53.4	125.7 ± 33.4	127.5 ± 32.9	130.0 ± 45.6
hsCRP (mg/L)	2.8 (1.5, 5.7)	3.1 (1.4, 6.4)	3.1 (1.3, 5.2)	2.9 (1.5, 5.9)
Sex (men)	71 (62)	68 (55)	65 (56)	71 (58)
Ethnicity, white	99 (86)	99 (80)	99 (85)	100 (82)
Current Smokers	14 (12)	16 (13)	12 (10)	13 (11)
Alcohol use				
none	14 (11)	12 (9)	4 (3)	7 (5)
<10 g/day	55 (44)	61 (46)	65 (52)	57 (43)
≥10–20 g/day	11 (9)	15 (11)	18 (14)	18 (14)
≥20 g/day	20 (16)	13 (10)	14 (11)	18 (14)
Education				
Low	16 (14)	30 (24)	23 (19)	32 (26)
Middle	70 (61)	62 (50)	69 (58)	60 (49)
High	14 (12)	9 (7)	8 (7)	9 (7)
Diabetes *	24 (21)	22 (18)	37 (32)	27 (22)
Obesity	29 (25)	31 (25)	33 (28)	28 (23)
Antihypertensive medication	98 (85)	95 (77)	95 (82)	93 (76)
ACE-inhibitor and/or ARB	68 (59)	52 (42)	71 (61)	66 (54)
Statins	81 (70)	78 (63)	79 (68)	81 (66)
Physically active				
No	8 (7)	10 (8)	11 (9)	11 (9)
Light active (<3 MET)	47 (40)	45 (36)	38 (32)	48 (39)
0–5 days moderate/vigorously active (≥3 MET)	31 (26)	30 (24)	29 (24)	33 (27)
≥5 days moderate/vigorously active (≥3 MET)	14 (12)	15 (12)	23 (19)	9 (7)

Data in Table 1 presented as mean ± SD, median (p25, p75) or % (*n*). * Diabetes was considered to be present if a patient reported having received the diagnosis from a physician, was taking antidiabetic drugs, or had an elevated plasma glucose level (≥7.0 mmol/L in the case of patients who had fasted more than 4 h or ≥11.1 mmol/L in the case of non-fasting patients). Antihypertensive medication ATC codes C02, C03, C07, C08 and C09. Obesity was defined as BMI > 30 kg/m^2^. Abbreviations: EPA, eicosapentaenoic acid; DHA, docosahexaenoic acid; ALA, alpha-linolenic acid; BMI, body mass index; BP, blood pressure; LDL, low-density lipoprotein; HDL, high-density lipoprotein; ACE, angiotensin converting enzyme; ARB, angiotensin receptor blocker; hsCRP, high sensitivity c-reactive protein; MET, metabolic equivalent tasks.

**Table 2:** Effect of 41 months intervention of omega-3 fatty acids on change in FGF23 levels in 336 patients of the Alpha Omega Trial according to study group with creatinine–cystatin C-based eGFR < 60 mL/min/1.73 m^2^.

	Pre-Treatment (95% CI) ^a^	Post-Treatment (95% CI) ^a^	Post-Treatment Adjusted for Pre-Treatment (95% CI) ^a^	Treatment Effect (95% CI) ^b^	*p*-Value ^c^
Placebo (*n* = 82)	159.0 (111.1, 206.9)	201.1 (146.8, 255.3) **^,d^	197.7 (141.5, 254.0)		
***Active intervention groups***					
EPA-DHA (*n* = 81)	179.1 (108.7, 249.5)	191.0 (152.0, 230.0) **	180.3 (123.6, 237.0)	−17 (−97, 62)	0.7
ALA (*n* = 86)	136.5 (115.9, 157.0)	229.2 (157.8, 300.7) ***	234.1 (179.2, 289.1)	36 (−42, 115)	0.4
EPA-DHA plus ALA (*n* = 87)	127.1 (103.2, 151.1)	223.6 (162.0, 285.2) ***	231.9 (177.2, 286.6)	34 (−44, 113)	0.4

^a^ Values are means (95% confidence interval of the mean), obtained by analysis of covariance (ANCOVA); ^b^ % effect of active treatment compared with placebo with 95% CI; ^c^ *p*-Values compared with placebo obtained by ANCOVA; ^d^ *p*-values obtained from paired *t*-test to check whether increases in FGF23 concentrations from pre-to posttreatment were statistically significant. * *p* < 0.05, ** *p* < 0.01, *** *p* < 0.001 vs. pre-treatment. Abbreviations: ALA: alpha-linoleic acid; DHA: docosahexaenoic acid; eGFR: estimated glomerular filtration rate; EPA: eicopentaenoic acid; FGF23: fibroblast growth factor.

**Table 3:** Effect of 41 months intervention of omega-3 fatty acids on change in FGF23 levels in 366 patients of the Alpha Omega Trial with creatinine–cystatin C-based eGFR < 60 mL/min/1.73 m^2^, according to 2 × 2 factorial design.

	Pre-Treatment (95% CI) ^a^	Post-Treatment (95% CI) ^a^	Post-Treatment Adjusted for Pre-Treatment (95% CI) ^a^	Treatment Effect (95% CI) ^b^	*p*-Value ^c^
n-3 fatty acids group combined vs. placebo (*n* = 254)	146.9 (122.2, 171.6)	215.1 (181.1, 249.1)	216.2 (184.2, 248.1)	18 (−46, 83)	0.6
ALA or combination of EPA-DHA and ALA vs. placebo or EPA-DHA only (*n* = 173 vs. 163) ^d^	131.8 (116.1, 147.4)	226.4 (179.7, 273.0)	233.0 (194.3, 271.7)	44 (−12, 100)	0.1
EPA-DHA or combination of EPA-DHA and ALA vs. placebo or ALA only (*n* = 168 vs. 168) ^d^	152.2 (116.2, 188.1)	207.9 (171.2, 244.6)	207.1 (167.7, 246.4)	−9 (−64, 46)	0.7

^a^ Values are means (95% confidence interval of the mean), obtained by analysis of covariance (ANCOVA); ^b^ % effect of active treatment vs. comparator group(s) as indicated with 95% CI; ^c^ *p*-Values compared with placebo obtained by ANCOVA; ^d^ According to the 2 × 2 factorial design the two groups that received ALA were combined and compared with the two groups that did not receive ALA. Similarly, the two groups that received EPA-DHA were combined and compared with the two groups that did not receive EPA-DHA. Abbreviations: ALA: alpha-linoleic acid; DHA: docosahexaenoic acid; EPA: eicopentaenoic acid; FGF23: fibroblast growth factor.
